# Synthesis, Characterization and Fluorescent Property Evaluation of 1,3,5-Triaryl-2-pyrazolines

**DOI:** 10.3390/molecules16097789

**Published:** 2011-09-13

**Authors:** Aurangzeb Hasan, Asghar Abbas, Muhammed Nadeem Akhtar

**Affiliations:** 1 Department of Chemistry, Faculty of Science, University of Malaya, Kuala Lumpur-50603, Malaysia; 2 Department of Chemistry, Faculty of Science, Quaid-i-Azam University, Islamabad 45320, Pakistan; 3 Laboratory of Natural Products, Institute of Bioscience, University Putra Malaysia, Kuala Lumpur 43400, Malaysia

**Keywords:** triarylpyrazolines, alkoxychalcones, synthesis, fluorescence

## Abstract

A series of 1,3,5-triaryl-2-pyrazolines was synthesized by dissolving the corresponding 4-alkoxychalcones in glacial acetic acid containing a few drops of concentrated hydrochloric acid. This step was followed by the addition of (3,4-dimethylphenyl) hydrazaine hydrochloride. Finally the target compounds were precipitated by pouring the reaction mixture onto crushed ice. The structures of the synthesized compounds were established by physicochemical and spectroscopic methods. The 1,3,5-triaryl-2-pyrazolines bearing homologous alkoxy groups were found to possess fluorescence properties in the blue region of the visible spectrum when irradiated with ultraviolet radiation. The fluorescent behavior of these compounds was studied by UV-Vis and emission spectroscopy, performed at room temperature.

## 1. Introduction

Due to the interesting properties of substituted pyrazolines as biological agents and optical materials, considerable attention has been focused on the study of this class of compounds. They have pharmaceutical importance as these can be effectively utilized as potential therapeutic agents against a variety of major ailments, due to their antibacterial and antifungal [1], antimicrobial [2,3] cytotoxic [4] anticancer [5] anti-inflammatory [6] and platelet aggregation inhibiting [7] properties. 1,3,5-Triaryl-2-pyrazolines constitute an important class of conjugated nitrogen-containing fluorescent compounds emitting a blue fluorescence with high quantum yield [8,9,10,11] and have been found to act as hole transporting media in photoconductive as well as emitting materials [12,13,14,15,16] and in organic electroluminescent devices (OELDs) [17,18,19,20]. Organic electroluminescent devices find potential use in various displays [21,22,23], and have many advantages over inorganic ones, such as high luminous efficiency, low cost, wide range of emission colors *via* specialized molecular design of organic compounds, and easy processing. 1,3,5-Triaryl-2-pyrazolines are also utilized as optical brightening agents for textiles, fabrics, plastics, papers [24] fluorescent switches [25] and as fluorescent probes in many chemosensors [26].

**Scheme 1:**
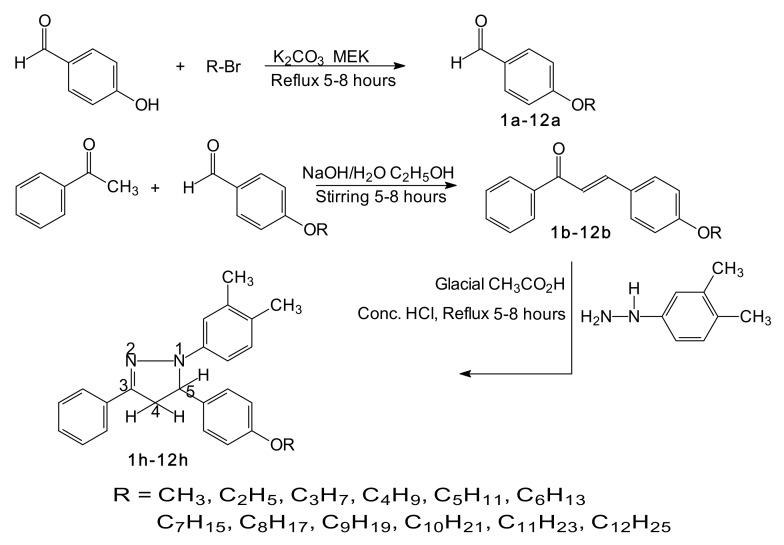
Synthesis of 1,3,5-triaryl-2-pyrazolines.

In this work we report the synthesis of some new 1,3,5-triaryl-2-pyrazolines **1h-12h** by converting benzaldehyde into the corresponding 4-alkoxybenzaldehydes **1a-12a** by treatment with appropriate alkyl halides. The 4-alkoxybenzaldehydes yielded 4-alkoxychalcones **1b-12b** on reaction with acetophenones in the presence of sodium hydroxide, water and ethanol. The target compounds were finally obtained by the reaction of 4-alkoxychalcones with (3,4-dimethylphenyl) hydrazaine hydrochloride (Scheme 1). 

## 2. Results and Discussion 

Twelve alkoxybenzaldehydes were synthesized by refluxing 4-hydroxybenzaldehyde with twelve alkyl bromides (CH_3_-Br – C_12_H_25_-Br) in the presence of potassium carbonate and butanone. The resulting 4-alkoxybenzaldehydes **1a-12a** were reacted with acetophenone in the presence of sodium hydroxide and ethanol to yield corresponding 4-alkoxychalcones **1b-12b**. 4-Alkoxychalcones were dissolved in glacial acetic acid containing a few drops of concentrated hydrochloric acid. This step was followed by the addition of (3,4-dimethylphenyl)hydrazaine hydrochloride. Finally the target compounds **1h-12h** were precipitated by pouring the reaction mixture onto crushed ice as outlined in Scheme 1.

The FT−IR spectra of synthesized 4-alkoxybenzaldehydes **1a−12a** showed two strong bands at stretching frequencies in the range of 1260–1245 cm^−1^ and 1050–1035 cm^−1^, which are characteristic of Ar−O−R groups. No peak appeared in the range of 3400–3300 cm^−1^, which indicated the disappearance of the –OH group of the 4-hydroxybenzaldehyde. A strong carbonyl group (C=O) band appeared in the 1700–1660 cm^−1^ range.

The 4-alkoxychalcones **1b−12b** were synthesized by base catalysed Claisen−Schmidt aldol condensation and were obtained as pale yellow crystalline solids. The FT-IR spectra of all the synthesized 4-alkoxychalcones showed absorption bands in the range of 1700–1675 cm^−1^ for carbonyl groups (C=O) and 1644–1617 cm^−1^ for olefinic double bonds (−CH=CH−). In addition the two strong characteristic Ar−O−R group bands were observed. 

Two prominent doublets δ = 7.37–7.43 ppm, *J* = 15.6 Hz and δ =7.80–7.81 ppm, *J* = 15.6 Hz appeared in the ^1^H-NMR spectra of 4-alkoxychalcones, which corresponds to the α- and β-hydrogen atoms, respectively, attached to the olefinic double bond of the α,β-unsaturated carbonyl system. 

The chemical shifts of doublets corresponding to the α-hydrogen atoms were found to be 7.37–7.43 ppm, with coupling constants of 15.6 Hz. The chemical shifts of doublets corresponding to the β-hydrogen atoms appeared at 7.80–7.81 ppm, with coupling constants of 15.6 Hz. The large coupling constant values confirmed that the 4-alkoxychalcones possess *trans* (*E*)-configurations. The aromatic protons of the 4-alkoxychalcones appeared downfield between 6.90 and 8.00 ppm in the aromatic region of the spectrum. The methylene protons (Ar−O−C***H***_2_−) of the alkoxy group adjacent to the oxygen atom appeared as a triplet. The chemical shifts of these protons were found between 3.90–4.10 ppm for all 4-alkoxychalcones, confirming the presence of ether (Ar−O−R) linkages. The chemical shifts of other aliphatic protons of the alkoxy group were found in the range of 0−2.0 ppm according to their respective splitting pattern.

The ^13^C-NMR of 4-alkoxychalcones **1b−12b** revealed carbonyl carbon peaks at 187–192 ppm. Aromatic carbon atom chemical shifts appeared in the range of 112–140 ppm. The aromatic carbon resonances of the compounds were assigned on the basis of signal intensities and comparison with the reported values. The ***C***_α_ and ***C***_β_ of the 4-alkoxychalcones were observed in the range of 118–121 ppm and 144–146 ppm, respectively. The methylene carbons attached to the oxygen atom of aromatic ring (ArO−***C***H_2_−) were found in the range of 60–70 ppm.

Gas chromatograms (GC) displayed single peaks with different retention times for every compound, confirming the purity of the compounds. Mass Spectrometry (MS) was used to determine the molecular weights of these compounds. A molecular ion peak (M^+^) was observed for the 4-alkoxychalcones **1b−12b**, in their respective mass spectra. The fragmentation pattern of synthesized compounds matched the typical pattern of chalcone fragmentation, further confirming the structures of the compounds.

The FT-IR spectra of the 1,3,5-triaryl-2-pyrazoline compounds **1h−12h**, showed absorption bands in the range of 1690–1640 cm^−1^ confirming the presence of carbon-nitrogen (C=N) double bonds, and at 1350–1000 cm^−1^ for carbon-nitrogen single bonds (C-N). A strong band for arylcarbon-alkylcarbon (Ar-R) appeared in the 1500–1490 cm^−1^ range. Two strong bands at stretching frequencies in the range of 1260–1245 cm^−1^ and 1050–1035 cm^−1^ were also observed, which confirmed the presence of Ar−O−R groups.

The formation of the five membered pyrazoline cycle was confirmed by the presence of two methylene protons (H*_a_* and H*_b_*) and one methine proton (H*_x_*) for the pyrazoline ring. The ^1^H-NMR spectra of the compounds **1h−12h** showed three doublet of doublets corresponding to the H*_a_*, H*_b_* and H*_x_* atoms of the pyrazoline ring, respectively. 

The chemical shifts of a doublet of doublets for *H_a_* of the compounds **1h−12h** were observed at 3.11 ppm, with coupling constants in the range of 7.5–7.8 Hz and 16.8–17.1 Hz. The chemical shifts of a doublet of doublets for *H_b_* of the compounds **1h−12h** were observed at 3.80 ppm with coupling constants in the range of 12.0–12.6 Hz and 16.8–17.1 Hz. The chemical shifts of a doublet of doublets for *H_x_* of the compounds **1h–12h** were observed at 5.20 ppm, with coupling constants in the range of 7.0–7.8, 12.0–12.6 Hz. The aromatic protons appeared downfield between 6.70 and 8.00 ppm with multiplicity according to the aromatic ring substitution pattern. The methylene protons (Ar−O−C***H***_2_−) of the alkoxy group adjacent to the oxygen atom appeared as a triplet. The chemical shifts for these protons are found around 3.90–4.10 ppm for all pyrazolines, confirming the presence of ether (Ar−O−C−) linkages. The chemical shifts of other aliphatic protons of the alkoxy groups were observed in the range of 0–2.0 ppm. The protons of methyl groups at the 3 and 4 positions of the aromatic ring directly attached to the nitrogen of the pyrazoline cycle in compounds **1h−12h** appeared as singlet in the range of 2.20–2.22 ppm and 2.16–2.17 ppm, respectively.

The proton-decoupled ^13^C-NMR spectra for all the synthesized 2-pyrazolines **1h−12h** were recorded at 75 MHz in CDCl_3_ and peak assignments were made on the basis of calculated and reported values [27,28]. The aromatic carbon resonances of the compounds are assigned on the basis of signal intensities and comparing with the calculated values. The methylene carbons attached to the oxygen atom of aromatic ring (ArO−***C***H_2_−) were observed around 60–70 ppm. In compounds **1h−12h**, the methylene carbon (−***C***H_2_−) and methine carbon (−***C***H−) of the five membered pyrazoline rings were assigned chemical shift values in the range of 41–44 ppm and 55–65 ppm, respectively.

**Figure 1:**
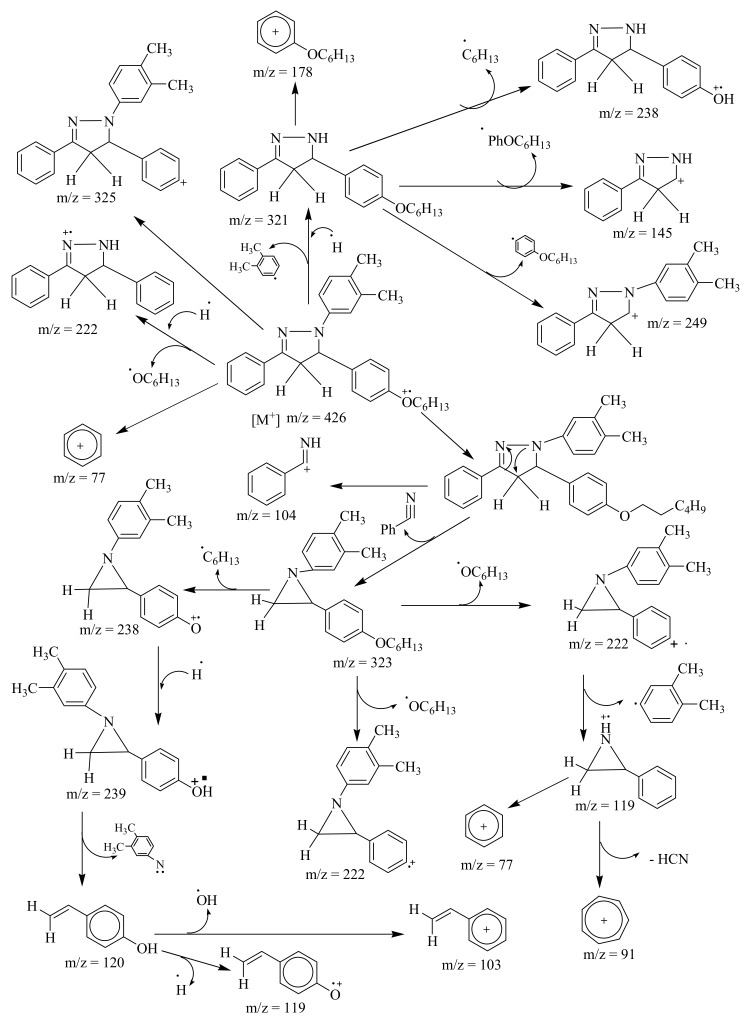
Mass fragmentation pattern for 1-(3,4-dimethylphenyl)-3-phenyl-5-(4-hexyloxyphenyl)-2-pyrazoline (**6h**).

**Figure 2:**
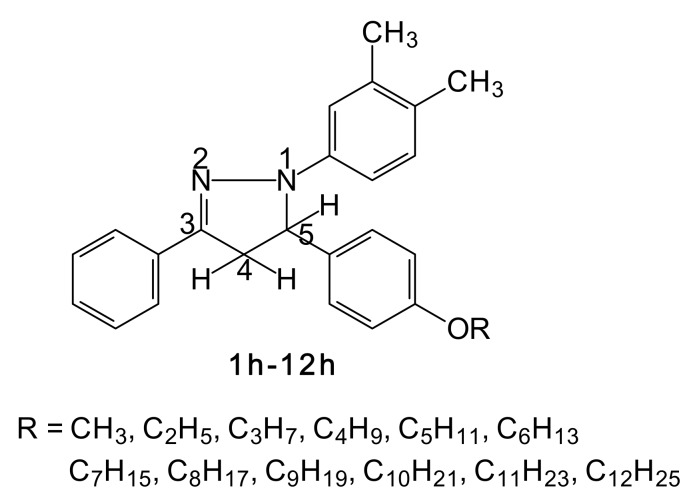
Structures of 1,3,5-triaryl-2-pyrazolines.

A molecular ion peak (M^+^) which was also the base peak was observed for all the synthesized 2-pyrazolines in their respective mass spectra. The fragmentation pattern of the synthesized compounds (exemplified by that of compound **6h**) matched the typical pattern of pyrazoline fragmentation [29] (Figure 1), confirming the structure of compounds. On the basis of the combined physical and spectral data, compounds 1,3,5-triaryl-2-pyrazolines were assigned the structures shown in Figure 2. 

The synthesized 1,3,5-triaryl-2-pyrazolines **1h−12h** bearing homologous alkoxy groups were found to possess fluorescence properties in the blue region of the visible spectrum when irradiated with ultra- violet radiation. The fluorescence behavior of these compounds was studied by UV-Vis and emission spectroscopy performed at room temperature.

**Figure 3:**
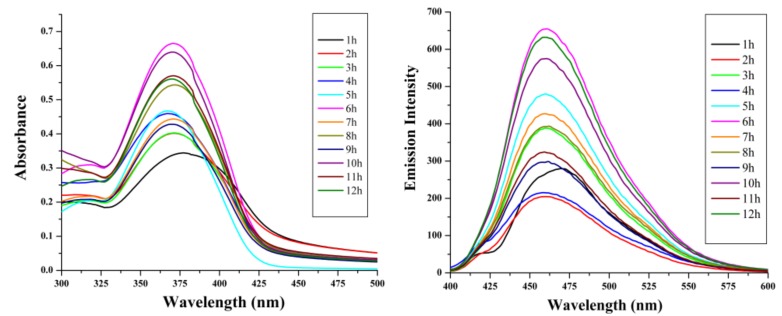
The UV-Vis absorption and emission spectra of **1h-12h**.

The UV-Vis spectra (Figure 3) of 1,3,5-triaryl-2-pyrazolines **1h−12h** exhibit an intense absorption band which is attributed to the π − π* transition of the conjugated backbone. In case of absorption spectra of compounds **1h−12h**, the absorption maxima (λ_max_^abs^) wavelength was observed in the range of 367−377 nm (Table 1) with the corresponding absorbance. The absorption spectra of the 1,3,5-triaryl-2-pyrazolines **1h−12h** showed that the spectral shapes are very similar because all these compounds possess the same (1,3,5-triaryl)-2-pyrazoline central nucleus. The difference of absorption wavelengths and absorption intensities is due to the effect of the presence of 4-alkoxyphenyl groups at the 5-position of the 2-pyrazoline moiety. In the emission spectra of 2-pyrazolines **1h−12h** (Figure 3) it was observed that all these compounds exhibited blue emission with variable emission intensity at constant concentration. The emission maxima (λ_max_^em^) wavelengths of all the synthesized compounds lie in the blue region of visible spectrum and therefore show blue fluorescence. 

The geometry of the 1,3,5-triaryl-2-pyrazoline nucleus has a configuration such that the aryl groups at the 1 and 3 positions are in conjugation with the pyrazoline ring. However, the aryl group at position 5 is not part of the conjugated moiety of the 2-pyrazoline nucleus. The alkoxy substitution at the aryl group at the 5 position can be expected to have some effect on the physico-chemical properties due to its electron donating nature. From emission spectra of the reported compounds, it can be concluded that the introduction of electron-donating 4-alkoxyphenyl groups at the 5-position strongly affects the emission intensity without causing any major blue- or red-shift in the emission wavelength. 

## 3. Experimental

### 3.1. General

Melting points of the synthesized compounds were recorded on a MFB-595-101M Gallenkamp digital melting point apparatus in open-end capillary tubes and are uncorrected. Thin layer chromatography was carried out on pre-coated silica gel plates (0.2 mm, E. Merck, 20 × 20 cm, 60F_254_). FT-IR spectra were recorded on a Bio-Red Merlin Spectrophotometer using KBr discs. ^1^H- NMR (300 MHz) and ^13^C-NMR (75.43 MHz) spectra were recorded on a Bruker AM-300 spectrometer in DMSO and CDCl_3_ solutions using TMS as internal standard. EIMS were recorded on a GCMS Agilent VG: 70 SE Mass Spectrometer. Purity of the synthesized compounds was also monitored by the same instrument.

### 3.2. Procedure for the Preparation of 4-Alkoxybenzaldehydes ***1a–12a***

4-Hydroxybenzaldehyde (0.1 mole, 12.2 g) and anhydrous K_2_CO_3_ (0.1 mole, 13.8 g) were added to 2-butanone (150 mL) in a 250 mL two neck round bottom flask. The reaction mixture was heated gently at 60–65 °C for 30 minutes, then 1-bromoalkane (Br-CH_3_ to Br-C_12_H_25_, 0.1 mole) was added drop wise and the reaction mixture was heated to reflux for 5–8 hours. The progress of the reaction was monitored by TLC. After completion of the reaction, the solvent was evaporated under reduced pressure on a rotary evaporator. The reaction mixture was transferred to a 1 L separatory funnel and diethyl ether (250 mL) was added. The K_2_CO_3_ was removed by washing the reaction mixture repeatedly with distilled water. Traces of unreacted 4-hydroxybenzaldehyde were removed by washing the ether layer with 5% NaOH solution (25 mL). The ether layer was washed with distilled water till it became neutral to pH paper and was dried on anhydrous MgSO_4_. The diethyl ether was evaporated to give the 4-alkoxybenzaldehydes **1a−12a** as transparent pale yellow liquids. 

### 3.2. Procedure for the Preparation of 4-Alkoxychalcones ***1b–12b***

In a 500 mL beaker acetophenone (0.1 mole, 12.0 g) was dissolved in ethanol (20 mL). To this mixture, ice cold 10% NaOH solution (200 mL) was added and the mixture stirred for 30 minutes at room temperature. Then 4-alkoxybenzaldehyde **1a–12a** (0.1 mole) was added to the reaction mixture that was further stirred for 5–8 hours till pale yellow precipitates appeared. Crushed ice was added to the solid mass and the reaction mixture was neutralized with dilute HCl. The products **1b–12b** were obtained as yellow solids, which were filtered and recrystallized from ethanol. 

### 3.3. Procedure for the Preparation of 1-(3,4-Dimethylphenyl)-3-Phenyl-5-(4-Alkoxyphenyl)-2-Pyrazolines ***1h–12h***

Synthesis of 1-(3,4-dimethylphenyl)-3-phenyl-5-(4-alkoxyphenyl)-2-pyrazolines was carried out by dissolving the 4-alkoxychalcones **1b–12b** (0.01 moles) in glacial acetic acid (25 mL) containing a few drops of concentrated hydrochloric acid and were heated to 60–65 °C for 30 minutes. (3,4-Dimethylphenyl)hydrazaine hydrochloride (0.02 mole, 3.45 g) was added to the reaction flask and the resulting mixture heated to reflux for 5–8 hours. The reaction mixture was cooled to room temperature and poured onto crushed ice to obtain the products. The precipitates obtained were filtered, washed with distilled water and dried in a desicator and were purified by column chromatography using silica gel and petroleum ether/ethyl acetate (4:1) as mobile phase. Purification of all the synthesized compounds was achieved by recrystallization and purity of each compound was monitored by thin layer (tlc) and gas (gc) chromatography.

*1-(3,4-Dimethylphenyl)-3-phenyl-5-(4-methoxyphenyl)-2-pyrazoline* (**1h**). 3-(4-Methoxyphenyl)-1-phenylprop-2-en-1-one (2.38 g, 10 mmol) and (3,4-dimethylphenyl) hydrazaine (2.72 g, 20 mmol) were reacted according to the general procedure. Color pale yellow solid; yield 80% (1.90 g); m.p. 120–124 °C; R_f_ = 0.88 (petroleum ether-ethyl acetate, 4:1), FT-IR (KBr, cm^−1^): 1682, 1292, 1497, 1257, 1047, ^1^H-NMR (300 MHz, CDCl_3_) δ 2.17 (s, 3H, N-Ar-4-CH_3_), 2.22 (s, 3H, N-Ar-3-CH_3_), 3.11 (dd, 1H, *J* = 7.8, 17.1 Hz, Ha), 3.75 (s, 3H, -O-CH_3_), 3.80 (dd, 1H, *J* = 12.3, 16.8 Hz, Hb), 5.20 (dd, 1H, *J* = 7.5, 12.3 Hz, Hx), 6.71 (d, 1H, *J* = 8.4 Hz, N-ArHh), 6.87 (d, 2H, *J* = 8.7 Hz, ArHc=c’), 6.93 (d, 1H, *J* = 8.4 Hz, N-ArHi), 7.06 (s, 1H, N-ArHj), 7.27 (d, 2H, *J* = 8.7 Hz, ArHd=d’), 7.33−7.43 (m, 3H, ArHf=f’, g), 7.74(d, 2H, *J* = 8.4 Hz, ArHe=e’), ^13^C-NMR (75 MHz, CDCl_3_) δ 18.8, 20.2, 43.5, 55.2, 64.2, 110.7, 114.4 (2C), 115.1, 125.6 (2C), 127.0 (2C), 128.3, 128.5 (2C), 129.8, 132.9, 134.9, 137.0, 143.1, 146.1, 158.8, 162.6 EIMS: m/z 356 (M+, base peak). Anal. Calcd. For C_24_H_24_N_2_O (Mol. Wt. 356): C, 80.87; H, 6.79; N, 7.86; Found: C, 80.81; H, 6.73; N, 7.92%.

*1-(3,4-Dimethylphenyl)-3-phenyl-5-(4-ethoxyphenyl)-2-pyrazoline* (**2h**). 3-(4-Ethoxyphenyl)-1-phenyl-prop-2-en-1-one (2.52 g, 10 mmol) and (3,4-dimethylphenyl) hydrazaine (2.72 g, 20 mmol) were reacted according to the general procedure. Color pale yellow solid; yield 85% (2.14 g); m.p. 102–106 °C; *R*_f_ = 0.85 (petroleum ether-ethyl acetate, 4:1), FT-IR (KBr, cm^−1^): 1685, 1297, 1495, 1255, 1043, ^1^H-NMR (300 MHz, CDCl_3_) *δ* 1.42 (t, 3H, *J* = 7.0 Hz, −O−CH*_2_*−C***H****_3_*), 2.17 (s, 3H, N- Ar-4-C*H*_3_), 2.22 (s, 3H, N-Ar-3-C*H*_3_), 3.11 (dd, 1H, *J* = 7.8, 17.1 Hz, *H_a_*), 3.80 (dd, 1H, *J* = 12.3, 16.8 Hz, *H_b_*), 4.02 (q, 2H, *J* = 7.2 Hz, −O−C***H****_2_*−), 5.20 (dd, 1H, *J* = 7.5, 12.3 Hz, *H_x_*), 6.72 (d, 1H, *J* = 8.4 Hz, N-Ar*H_h_*), 6.87 (d, 2H, *J* = 8.7 Hz, Ar*H_c=c’_*), 6.93 (d, 1H, *J* = 8.4 Hz, N- Ar*H_i_*), 7.06 (s, 1H, N-Ar*H_j_*), 7.25 (d, 2H, *J* = 8.7 Hz, Ar*H_d=d’_*), 7.31-7.43 (m, 3H, Ar*H_f=f’, g_*), 7.75(d, 2H, *J* = 8.4 Hz, Ar*H_e=e’_*), ^13^C-NMR (75 MHz, CDCl_3_) *δ* 14.8, 18.8, 20.2, 43.5, 63.4, 64.3, 110.7, 114.9, 115.1, 125.6 (2C), 127.0 (2C), 127.1, 128.3, 128.5, 129.8, 133.0, 134.8, 137.4, 143.2, 146.1, 158.2, EIMS: *m/z* 370 (M^+^, base peak). Anal. Calcd. For C_25_H_26_N_2_O (Mol. Wt. 370): C, 81.05; H, 7.07; N, 7.56; Found: C, 81.01; H, 7.02; N, 7.64%.

*1-(3,4-Dimethylphenyl)-3-phenyl-5-(4-propyloxyphenyl)-2-pyrazoline* (**3h**). 3-(4-Propyloxyphenyl)-1-phenylprop-2-en-1-one (2.66 g, 10 mmol) and (3,4-dimethylphenyl) hydrazaine (2.72 g; 20 mmol) were reacted according to the general procedure. Color pale yellow solid; yield 81% (2.15 g); m.p. 126–130 °C; *R*_f_ = 0.87 (petroleum ether-ethyl acetate, 4:1), FT-IR (KBr, cm^−1^): 1687, 1299, 1493, 1254, 1042, ^1^H-NMR (300 MHz, CDCl_3_) *δ* 1.04 (t, 3H, *J* = 7.2 Hz, −O−CH*_2_*−CH*_2_*−C***H****_3_*), 1.81 (sextet, 2H, *J* = 7.2 Hz, −O−CH*_2_*−C***H****_2_*−CH*_3_*), 2.17 (s, 3H, N-Ar-4-C***H***_3_), 2.22 (s, 3H, N-Ar-3-C***H***_3_), 3.11 (dd, 1H, *J* = 7.5, 16.8 Hz, *H_a_*), 3.80 (dd, 1H, *J* = 12.3, 16.8 Hz, *H_b_*), 3.90 (t, 2H, *J* = 6.6 Hz, −O−C***H****_2_*−), 5.20 (dd, 1H, *J* = 7.5, 12.3 Hz, *H_x_*), 6.72 (d, 1H, *J* = 8.4 Hz, N-Ar*H_h_*), 6.87 (d, 2H, *J* = 8.7 Hz, Ar*H_c=c’_*), 6.93 (d, 1H, *J* = 8.4 Hz, N-Ar*H_i_*), 7.06 (s, 1H, N-Ar*H_j_*), 7.25 (d, 2H, *J* = 8.7 Hz, Ar*H_d=d’_*), 7.33-7.43 (m, 3H, Ar*H_f= f’, g_*), 7.74(d, 2H, *J* = 8.4 Hz, Ar*H_e=e’_*); ^13^C-NMR (75 MHz, CDCl_3_) *δ* 10.5, 18.8, 20.2, 22.6, 43.6, 64.3, 69.4, 110.7, 114.9 (2C), 115.1, 125.6 (2C), 127.0 (2C), 127.1, 128.3, 128.5 (2C), 129.8, 133.0, 134.7, 137.0, 143.1, 146.1, 158.4, EIMS: *m/z* 384 (M^+^, base peak). Anal. Calcd. For C_26_H_28_N_2_O (Mol. Wt. 384): C, 81.21; H, 7.34; N, 7.29; Found: C, 81.16; H, 7.31; N, 7.37%.

*1-(3,4-Dimethylphenyl)-3-phenyl-5-(4-butyloxyphenyl)-2-pyrazoline* (**4h**). 3-(4-Butyloxyphenyl)-1-phenylprop-2-en-1-one (2.80 g, 10 mmol) and (3,4-dimethylphenyl) hydrazaine (2.72 g, 20 mmol) were reacted according to the general procedure. Color pale yellow solid; yield 86% (2.40 g); m.p. 122–125 °C; *R*_f_ = 0.87 (petroleum ether-ethyl acetate, 4:1), FT-IR (KBr, cm^−1^): 1680, 1294, 1494, 1258, 1052, ^1^H-NMR (300 MHz, CDCl_3_) *δ* 0.99 (t, 3H, *J* = 7.2 Hz, −O−(CH*_2_*)*_3_*−C***H****_3_*), 1.50 (sextet, 2H, *J* = 7.8 Hz, −O−CH_2_−CH_2_−C***H***_2_− CH*_3_*), 1.77 (qn, 2H, *J* = 8.0 Hz, −O−CH_2_−C***H***_2_−C_2_H*_5_*), 2.17 (s, 3H, N-Ar-4-C***H***_3_), 2.22 (s, 3H, N-Ar-3-C***H***_3_), 3.11 (dd, 1H, *J* = 7.5, 16.8 Hz, *H_a_*), 3.80 (dd, 1H, *J* = 12.3, 16.8 Hz, *H_b_*), 3.95 (t, 2H, *J* = 6.6 Hz, −O−C***H***_2_−), 5.20 (dd, 1H, *J* = 7.5, 12.3 Hz, *H_x_*), 6.72 (d, 1H, *J* = 8.0 Hz, N-Ar*H_h_*), 6.87 (d, 2H, *J* = 8.7 Hz, Ar*H_c=c’_*), 6.93 (d, 1H, *J* = 8.1 Hz, N-Ar*H_i_*), 7.06 (s, 1H, N-Ar*H_j_*), 7.25 (d, 2H, *J* = 8.7 Hz, Ar*H_d=d’_*), 7.33-7.43 (m, 3H, Ar*H_f=f’, g_*), 7.75(d, 2H, *J* = 8.4 Hz, Ar*H_e=e’_*); ^13^C-NMR (75 MHz, CDCl_3_) *δ* 13.9, 18.8, 19.2, 20.2, 31.3, 43.60, 64.3, 67.6, 110.7, 114.9 (2C), 115.1, 125.6 (2C), 127.0 (2C), 127.1, 128.3, 128.5 (2C), 129.8, 133.0, 134.7, 137.0, 143.1, 146.1, 158.4; EIMS: *m/z* 398 (M^+^, base peak). Anal. Calcd. For C_27_H_30_N_2_O (Mol. Wt. 398): C, 81.37; H, 7.59; N, 7.03; Found: C, 81.31; H, 7.55; N, 7.13%.

*1-(3,4-Dimethylphenyl)-3-phenyl-5-(4-pentyloxyphenyl)-2-pyrazoline* (**5h**). 3-(4-Pentyloxyphenyl)-1-phenylprop-2-en-1-one (2.94 g, 10 mmol) and (3,4-dimethylphenyl) hydrazaine (2.72 g, 20 mmol) were reacted according to the general procedure. Color pale yellow solid; yield 84% (2.46 g); m.p. 121–125 °C; *R*_f_ = 0.89 (petroleum ether-ethyl acetate, 4:1), FT-IR (KBr, cm^−1^): 1686, 1296, 1491, 1256, 1054, ^1^H-NMR (300 MHz, CDCl_3_) *δ* 0.94 (t, 3H, *J* = 7.2 Hz, −O−(CH*_2_*)*_4_*−C***H****_3_*), 1.38−1.46 (m, −O−CH_2_−CH_2_−(C***H***_2_)_2_− CH*_3_*), 1.78 (qn, 2H, *J* = 7.2 Hz, −O−CH_2_−C***H***_2_−C_3_H*_7_*), 2.17 (s, 3H, N-Ar-4-C*H*_3_), 2.22 (s, 3H, N-Ar-3-C*H*_3_) , 3.11 (dd, 1H, *J* = 7.8, 17.1 Hz, *H_a_*), 3.80 (dd, 1H, *J* = 12.3, 17.1 Hz, *H_b_*), 3.94 (t, 2H, *J* = 6.6 Hz, −O−C***H***_2_−), 5.20 (dd, 1H, *J* = 7.5, 12.3 Hz, *H_x_*), 6.72 (d, 1H, *J* = 8.1 Hz, N-Ar*H_h_*), 6.86 (d, 2H, *J* = 8.7 Hz, Ar*H_c=c’_*), 6.93 (d, 1H, *J* = 8.1 Hz, N-Ar*H_i_*), 7.06 (s, 1H, N-Ar*H_j_*), 7.25 (d, 2H, *J* = 8.7 Hz, Ar*H_d=d’_*), 7.32-7.43 (m, 3H, Ar*H_f=f’, g_*), 7.74(d, 2H, *J* = 8.1 Hz, Ar*H_e=e’_*); ^13^C-NMR (75 MHz, CDCl_3_) *δ* 14.0, 18.8, 20.2, 22.4, 28.2, 28.9, 43.6, 64.3, 67.9, 110.7, 114.9, 115.1, 125.6, 127.0, 127.1, 128.3, 128.5, 129.8, 133.0, 134.7, 137.0, 143.1, 146.1, 158.4; EIMS: *m/z* 412 (M^+^, base peak). Anal. Calcd. For C_28_H_32_N_2_O (Mol. Wt. 412): C, 81.51; H, 7.82; N, 6.79; Found: C, 81.48; H, 7.79; N, 6.85%.

*1-(3,4-Dimethylphenyl)-3-phenyl-5-(4-hexyloxyphenyl)-2-pyrazoline* (**6h**). 3-(4-Hexyloxyphenyl)-1-phenylprop-2-en-1-one (3.08 g, 10 mmol) and (3,4-dimethylphenyl) hydrazaine (2.72 g, 20 mmol) were reacted according to the general procedure. Color pale yellow solid; yield 87% (2.67 g); m.p. 88–93 °C *R*_f_ = 0.87 (petroleum ether-ethyl acetate, 4:1), FT- IR (KBr, cm^−1^): 1679, 1292, 1497, 1258, 1048, ^1^H-NMR (300 MHz, CDCl_3_) *δ* 0.92 (t, 3H, *J* = 7.2 Hz, −O−(CH*_2_*)*_5_*−C***H****_3_*), 1.32−1.48 (m, 6H, −O−CH_2_−CH_2_−(C***H***_2_)_3_− CH*_3_*), 1.78 (qn, 2H, *J* = 7.2 Hz, −O−CH_2_−C***H***_2_−C_4_H*_9_*), 2.16 (s, 3H, N-Ar-4- C***H***_3_), 2.22 (s, 3H, N-Ar-3-C***H***_3_), 3.11 (dd, 1H, *J* = 7.5, 16.8 Hz, ***H****_a_*), 3.80 (dd, 1H, *J* = 12.3, 17.1 Hz, ***H****_b_*), 3.93 (t, 2H, *J* = 6.6 Hz, −O−CH_2_−), 5.20 (dd, 1H, *J* = 7.0, 12.0 Hz, ***H****_x_*), 6.71 (d, 1H, *J* = 8.1 Hz, N- Ar***H****_h_*), 6.86 (d, 2H, *J* = 8.7 Hz, Ar***H****_c=c’_*), 6.93 (d, 1H, *J* = 8.1 Hz, N-Ar***H****_i_*), 7.06 (s, 1H, N-Ar***H****_j_*), 7.25 (d, 2H, *J* = 8.7 Hz, Ar***H****_d=d’_*), 7.32-7.43 (m, 3H, Ar***H****_f=f’, g_*), 7.74(d, 2H, *J* = 8.1 Hz, Ar***H****_e=e’_*); ^13^C-NMR (75 MHz, CDCl_3_) *δ* 14.0, 18.8, 20.2, 22.6, 25.7, 29.2, 31.6, 43.6, 64.3, 68.0, 110.7, 114.9, 115.1, 125.6, 127.0, 127.1, 128.3, 128.5, 129.8, 133.0, 134.7, 137.0, 143.1, 146.1, 158.4; EIMS: *m/z* 426 (M^+^, base peak). Anal. Calcd. For C_29_H_34_N_2_O (Mol. Wt. 426): C, 81.65; H, 8.03; N, 6.57; Found: C, 81.65; H, 8.03; N, 6.57%.

*1-(3, 4-Dimethylphenyl)-3-phenyl-5-(4-heptyloxyphenyl)-2-pyrazoline* (**7h**). 3*-(*4*-*Heptyloxyphenyl)-1-phenylprop-2-en-1-one (3.22 g, 10 mmol) and (3,4-dimethylphenyl) hydrazaine (2.72 g, 20 mmol) were reacted according to the general procedure. Color pale yellow solid; yield 83% (2.67 g); m.p. 93–97 °C; *R*_f_ = 0.85 (petroleum ether-ethyl acetate, 4:1), FT-IR (KBr, cm^−1^): 1677, 1294, 1488, 1257, 1043, ^1^H-NMR (300 MHz, CDCl_3_) *δ* 0.91 (t, 3H, *J* = 7.2 Hz, −O−(CH*_2_*)*_6_*−C***H****_3_*), 1.31−1.48 (m, 8H, −O−CH_2_−CH_2_−(C***H***_2_)_4_− CH*_3_*), 1.77 (qn, 2H, *J* = 7.8 Hz, −O−CH_2_−C***H***_2_−C_5_H*_11_*), 2.16 (s, 3H, N-Ar-4-C*H*_3_), 2.21 (s, 3H, N-Ar-3-C*H*_3_), 3.11 (dd, 1H, *J* = 7.8, 17.1 Hz, *H_a_*), 3.80 (dd, 1H, *J* = 12.3, 17.1 Hz, *H_b_*), 3.93 (t, 2H, *J* = 6.6 Hz, −O−CH_2_−), 5.20 (dd, 1H, *J* = 7.5, 12.3 Hz, *H_x_*), 6.71 (d, 1H, *J* = 8.4 Hz, N-Ar*H_h_*), 6.86 (d, 2H, *J* = 8.7 Hz, Ar*H_c=c’_*), 6.92 (d, 1H, *J* = 8.1 Hz, N-Ar*H_i_*), 7.05 (s, 1H, N-Ar*H_j_*), 7.24 (d, 2H, *J* = 8.7 Hz, Ar*H_d=d’_*), 7.32-7.42 (m, 3H, Ar*H_f=f’, g_*), 7.74(d, 2H, *J* = 8.1 Hz, Ar*H_e=e’_*); ^13^C-NMR (75 MHz, CDCl_3_) *δ* 14.1, 18.8, 20.1, 22.6, 26.0, 29.2, 31.7, 34.7, 43.5, 64.3, 68.0, 110.7, 114.9, 115.1, 125.6, 127.0, 127.1, 128.3, 128.5, 129.8, 133.0, 134.7, 137.0, 143.1, 146.1, 158.4; EIMS: *m/z* 440 (M^+^, base peak). Anal. Calcd. For C_30_H_36_N_2_O (Mol. Wt. 440): C, 81.78; H, 8.24; N, 6.36; Found: C, 81.72; H, 8.18; N, 6.43%.

*1-(3,4-Dimethylphenyl)-3-phenyl-5-(4-octyloxyphenyl)-2-pyrazoline* (**8h**). 3-(4-Octyloxyphenyl)-1-phenylprop-2-en-1-one (3.36 g, 10 mmol) and (3,4-dimethylphenyl) hydrazaine (2.72 g, 20 mmol) were reacted according to the general procedure. Color pale yellow solid; yield 86% (2.88 g); m.p. 96–99 °C; *R*_f_ = 0.87 (petroleum ether-ethyl acetate, 4:1), FT-IR (KBr, cm^−1^): 1685, 1298, 1491, 1253, 1057, ^1^H-NMR (300 MHz, CDCl_3_) *δ* 0.91 (t, 3H, *J* = 7.2 Hz, −O−(CH*_2_*)*_7_*−C***H****_3_*), 1.30−1.45 (m, 10H, −O−CH_2_−CH_2_−(C***H***_2_)_5_− CH*_3_*), 1.78 (qn, 2H, *J* = 8.0 Hz, −O−CH_2_−C***H***_2_−C_6_H*_13_*), 2.16 (s, 3H, N-Ar-4- C*H*_3_), 2.21 (s, 3H, N-Ar-3-C*H*_3_), 3.11 (dd, 1H, *J* = 7.8, 17.1 Hz, *H_a_*), 3.80 (dd, 1H, *J* = 12.3, 17.1 Hz, *H_b_*), 3.93 (t, 2H, *J* = 6.6 Hz, −O−CH_2_−), 5.20 (dd, 1H, *J* = 7.5, 12.0 Hz, *H_x_*), 6.71 (d, 1H, *J* = 8.1 Hz, N-Ar*H_h_*), 6.86 (d, 2H, *J* = 8.7 Hz, Ar*H_c=c’_*), 6.93 (d, 1H, *J* = 8.1 Hz, N-Ar*H_i_*), 7.06 (s, 1H, N-Ar*H_j_*), 7.25 (d, 2H, *J* = 8.7 Hz, Ar*H_d=d’_*), 7.32–7.45 (m, 3H, Ar*H_f=f’, g_*), 7.74(d, 2H, *J* = 8.4 Hz, Ar*H_e=e’_*); ^13^C- NMR (CDCl_3_) *δ* 14.1, 18.8, 20.2, 22.6, 26.0, 29.2, 29.3, 31.8, 35.5, 43.5, 64.3, 67.9, 110.7, 114.9 (2C), 115.1, 125.6 (2C), 127.0 (2C), 127.1, 128.3, 128.5 (2C), 129.8, 132.9, 134.7, 137.0, 143.1, 146.1, 158.4; EIMS: *m/z* 454 (M^+^, base peak). Anal. Calcd. For C_31_H_38_N_2_O (Mol. Wt. 454): C, 81.89; H, 8.42; N, 6.16; Found: C, 81.85; H, 8.38; N, 6.25%.

*1-(3,4-Dimethylphenyl)-3-phenyl-5-(4-nonyloxyphenyl)-2-pyrazoline* (**9h**). 3-(4-Nonyloxyphenyl)-1-phenylprop-2-en-1-one (3.50 g, 10 mmol) and (3,4-dimethylphenyl) hydrazaine (2.72 g, 20 mmol) were reacted according to the general procedure. Color pale yellow solid; yield 85% (2.97 g); m.p. 101–103 °C; *R*_f_ = 0.85 (petroleum ether-ethyl acetate, 4:1), FT-IR (KBr, cm^−1^): 1679, 1292, 1493, 1254, 1055, ^1^H-NMR (300 MHz, CDCl_3_) *δ* 0.90 (t, 3H, *J* = 7.2 Hz, −O−(CH*_2_*)*_8_*−C***H****_3_*), 1.30−1.47 (m, 12H, −O−CH_2_−CH_2_−(C***H***_2_)_6_− CH*_3_*), 1.78 (qn, 2H, *J* = 7.8 Hz, −O−CH_2_−C***H***_2_−C_7_H*_15_*), 2.17 (s, 3H, N-Ar-4- C*H*_3_), 2.22 (s, 3H, N-Ar-3-C*H*_3_), 3.11 (dd, 1H, *J* = 7.5, 16.8 Hz, *H_a_*), 3.80 (dd, 1H, *J* = 12.3, 17.1 Hz, *H_b_*), 3.93 (t, 2H, *J* = 6.6 Hz, −O−CH_2_−), 5.20 (dd, 1H, *J* = 7.5, 12.3 Hz, *H_x_*), 6.71 (d, 1H, *J* = 8.1 Hz, N-Ar*H_h_*), 6.87 (d, 2H, *J* = 8.7 Hz, Ar*H_c=c’_*), 6.93 (d, 1H, *J* = 8.1 Hz, N-Ar*H_i_*), 7.06 (s, 1H, N-Ar*H_j_*), 7.25 (d, 2H, *J* = 8.7 Hz, Ar*H_d=d’_*), 7.33-7.43 (m, 3H, Ar*H_f=f’, g_*), 7.75(d, 2H, *J* = 8.4 Hz, Ar*H_e=e’_*); ^13^C-NMR (75 MHz, CDCl_3_) *δ* 14.1, 18.8, 20.2, 22.7, 26.0, 29.3 (2C), 29.4, 29.5, 31.9, 43.6, 64.3, 67.9, 110.7, 114.9, 115.1, 125.6, 127.0, 127.1, 128.3, 128.5, 129.8, 133.0, 134.7, 137.0, 143.1, 146.1, 158.4; EIMS: *m/z* 468 (M^+^, base peak). Anal. Calcd. For C_32_H_40_N_2_O (Mol. Wt. 468): C, 82.01; H, 8.60; N, 5.98; Found: C, 81.97; H, 8.57; N, 6.05%.

*1-(3,4-Dimethylphenyl)-3-phenyl-5-(4-decyloxyphenyl)-2-pyrazoline* (**10h**). 3-(4-Decyloxyphenyl)-1-phenylprop-2-en-1-one (3.64 g, 10 mmol) and (3,4-dimethylphenyl) hydrazaine (2.72 g, 20 mmol) were reacted according to the general procedure. Color pale yellow solid; yield 81% (2.94 g); m.p. 85–87 °C; *R*_f_ = 0.86 (petroleum ether-ethyl acetate, 4:1), FT-IR (KBr, cm^−1^): 1687, 1297, 1498, 1259, 1049, ^1^H-NMR (300 MHz, CDCl_3_) *δ* 0.90 (t, 3H, *J* = 7.2 Hz, −O−(CH*_2_*)*_9_*−C***H****_3_*), 1.30−1.47 (m, 14H, −O−CH_2_−CH_2_−(C***H***_2_)_7_− CH*_3_*), 1.78 (qn, 2H, *J* = 7.2 Hz, −O−CH_2_−C***H***_2_−C_8_H*_17_*), 2.16 (s, 3H, N-Ar-4- C*H*_3_), 2.20 (s, 3H, N-Ar-3-C*H*_3_), 3.11 (dd, 1H, *J* = 7.5, 17.1 Hz, *H_a_*), 3.80 (dd, 1H, *J* = 12.3, 17.1 Hz, *H_b_*), 3.93 (t, 2H, *J* = 6.6 Hz, −O−CH_2_−), 5.20 (dd, 1H, *J* = 7.5, 12.0 Hz, *H_x_*), 6.71 (d, 1H, *J* = 8.1 Hz, N-Ar*H_h_*), 6.86 (d, 2H, *J* = 8.7 Hz, Ar*H_c=c’_*), 6.92 (d, 1H, *J* = 8.1 Hz, N-Ar*H_i_*), 7.06 (s, 1H, N-Ar*H_j_*), 7.25 (d, 2H, *J* = 8.7 Hz, Ar*H_d=d’_*), 7.32-7.47 (m, 3H, Ar*H_f=f’, g_*), 7.74(d, 2H, *J* = 8.4 Hz, Ar*H_e=e’_*); ^13^C- NMR (75 MHz, CDCl_3_) *δ* 14.1, 18.8, 20.2, 22.7, 26.0, 29.2, 29.3, 29.4, 29.5, 29.6, 31.9, 43.5, 64.3, 68.0, 110.7, 114.9 (2C), 115.1, 125.6 (2C), 127.0, 127.1, 128.3, 128.5 (2C), 129.8, 133.0, 134.7, 137.0, 143.1, 146.1, 158.4; EIMS: *m/z* 482 (M^+^, base peak). Anal. Calcd. For C_33_H_42_N_2_O (Mol. Wt. 482): C, 82.11; H, 8.77; N, 5.80; Found: C, 82.09; H, 8.74; N, 5.88%.

*1-(3,4-Dimethylphenyl)-3-phenyl-5-(4-undecyloxyphenyl)-2-pyrazoline* (**11h**). 3-(4-Unecyloxyphenyl)-1-phenylprop-2-en-1-one (3.76 g, 10 mmol) and (3,4-dimethylphenyl) hydrazaine (2.72 g, 20 mmol) were reacted according to the general procedure. Color pale yellow solid; yield 87% (3.27 g); m.p. 88–92 °C; *R*_f_ = 0.85 (petroleum ether-ethyl acetate, 4:1), FT-IR (KBr, cm^−1^): 1689, 1295, 1494, 1256, 1054, ^1^H-NMR (300 MHz, CDCl_3_) *δ* 0.90 (t, 3H, *J* = 7.2 Hz, −O−(CH*_2_*)*_10_*−C***H****_3_*), 1.29−1.47 (m, 16H, −O−CH_2_−CH_2_−(C***H***_2_)_8_− CH*_3_*), 1.78 (qn, 2H, *J* = 8.0 Hz, −O−CH_2_−C***H***_2_−C_9_H*_19_*), 2.17 (s, 3H, N-Ar-4- C*H*_3_), 2.22 (s, 3H, N-Ar-3-C*H*_3_), 3.11 (dd, 1H, *J* = 7.8, 17.1 Hz, *H_a_*), 3.80 (dd, 1H, *J* = 12.3, 17.1 Hz, *H_b_*), 3.93 (t, 2H, *J* = 6.6 Hz, −O−CH_2_−), 5.20 (dd, 1H, *J* = 7.5, 12.3 Hz, *H_x_*), 6.71 (d, 1H, *J* = 8.1 Hz, N-Ar*H_h_*), 6.86 (d, 2H, *J* = 8.7 Hz, Ar*H_c=c’_*), 6.93 (d, 1H, *J* = 8.1 Hz, N-Ar*H_i_*), 7.06 (s, 1H, N-Ar*H_j_*), 7.25 (d, 2H, *J* = 8.7 Hz, Ar*H_d=d’_*), 7.32-7.45 (m, 3H, Ar*H_f=f’, g_*), 7.74(d, 2H, *J* = 7.8 Hz, Ar*H_e=e’_*); ^13^C- NMR (75 MHz, CDCl_3_) *δ* 14.1, 18.8, 20.2, 22.7, 26.0, 29.3, 29.3, 29.4, 29.6, 29.7, 31.9, 43.6, 64.3, 67.9, 110.7, 114.3, 114.9 (2C), 115.1, 125.6 (2C), 127.0 (2C), 127.1, 128.3, 128.5, (2C), 129.8, 132.9, 134.7, 137.0, 143.1, 146.1, 158.4; EIMS: *m/z* 496 (M^+^, base peak). Anal. Calcd. For C_34_H_44_N_2_O (Mol. Wt. 496): C, 82.21; H, 8.93; N, 5.64; Found: C, 82.17; H, 8.89; N, 5.71%.

*1-(3,4-Dimethylphenyl)-3-phenyl-5-(4-dodecyloxyphenyl)-2*-*pyrazoline*(**12h**). 3-(4-Dodecyloxyphenyl)-1-phenylprop-2-en-1-one (3.90 g, 10 mmol) and (3,4-dimethylphenyl) hydrazaine (2.72 g, 20 mmol) were reacted according to the general procedure. Color pale yellow solid; yield 84% (3.27 g); m.p. 86–88 °C; *R*_f_ = 0.87 (petroleum ether-ethyl acetate, 4:1), FT-IR (KBr, cm^−1^): 1684, 1293, 1493, 1253, 1052, ^1^H-NMR (300 MHz, CDCl_3_) *δ* 0.90 (t, 3H, *J* = 7.2 Hz, −O−(CH*_2_*)*_11_*−C***H****_3_*), 1.29−1.47 (m, 18H, −O−CH_2_−CH_2_−(C***H***_2_)_9_− CH*_3_*), 1.78 (qn, 2H, *J* = 7.8 Hz, −O−CH_2_−C***H***_2_−C_10_H*_21_*), 2.17 (s, 3H, N-Ar-4-C***H***_3_), 2.22 (s, 3H, N-Ar-3-C***H***_3_), 3.11 (dd, 1H, *J* = 7.5, 16.8 Hz, *H_a_*), 3.80 (dd, 1H, *J* = 12.3, 17.1 Hz, *H_b_*), 3.93 (t, 2H, *J* = 6.6 Hz, –O–C*H*_2_–), 5.20 (dd, 1H, *J* = 7.8, 12.3 Hz, *H_x_*), 6.72 (d, 1H, *J* = 8.1 Hz, N-Ar*H_h_*), 6.87 (d, 2H, *J* = 8.7 Hz, Ar*H_c=c’_*), 6.93 (d, 1H, *J* = 8.1 Hz, N-Ar*H_i_*), 7.06 (s, 1H, N- Ar*H_j_*), 7.25 (d, 2H, *J* = 8.7 Hz, Ar*H_d=d’_*), 7.32-7.43 (m, 3H, Ar*H_f=f’, g_*), 7.74(d, 2H, *J* = 8.4 Hz, Ar*H_e=e’_*); ^13^C-NMR (75 MHz, CDCl_3_) *δ* 14.1, 18.8, 20.2, 26.0, 29.3, 29.3, 29.4, 29.6, 29.6, 29.6, 29.6, 31.9, 43.6, 64.3, 68.0, 110.7, 114.9 (2C), 115.1, 125.6 (2C), 127.0 (2C), 127.1, 128.3, 128.5 (2C), 129.8, 133.0, 134.7, 137.0, 143.1, 146.1, 158.4; EIMS: *m/z* 510 (M^+^, base peak). Anal. Calcd. For C_35_H_46_N_2_O (Mol. Wt. 510): C, 82.30; H, 9.08; N, 5.48; Found: C, 82.24; H, 9.01; N, 5.54%.

### 3.4. Fluorescence Properties of 1,3,5-Triaryl-2-pyrazolines 

The fluorescent behavior of these compounds was studied by UV-Vis and emission spectroscopy, performed at room temperature (298 K). The UV-Vis and the emission spectra of 1,3,5-triaryl-2-pyrazolines **1h−12h**, were recorded in *N,N*-dimethylformamide-water (3:7) solution at a concentration of 1 × 10^−5^ mol L^−1^ and 1 × 10^−7^ mol L^−1^, respectively.

## 4. Conclusions 

A series of twelve 1,3,5-triaryl-2-pyrazolines **1h-12h** containing homologous alkoxy groups (CH_3_-C_12_H_25_) were synthesized by converting benzaldehyde into 4-alkoxybenzaldehydes with the corresponding alkyl halides. The 4-alkoxybenzaldehydes yielded 4-alkoxychalcones on reaction with acetophenone. The target compounds were finally obtained by the reaction of the 4-alkoxychalcones with (3,4-dimethylphenyl) hydrazaine hydrochloride. Their structures were confirmed by IR, ^1^H- and ^13^C–NMR, mass spectrometric and elemental analysis. The synthesized 1,3,5-triaryl-2-pyrazolines were found to possess fluorescence properties in the blue region of the visible spectrum when irradiated with ultraviolet radiation. The comparison of emission intensities of pyrazoline derivatives revealed that the alkoxy chains have remarkable effect on emission intensity of the 1,3,5-triaryl-2-pyrazolines. The absorption intensities of **6h**, **10h 11h**, **12h** and **8h** are much higher than other derivatives. Approximately, it was the same case in emission intensities. It can thus be concluded that variation in alkoxy chains can be used to tune the emission intensity that can find potential applications in controlling/optimizing the optoelectronic and luminescence properties of Organic Light Emitting Diodes (OLEDs) based on such types of fluorescent compounds. 

## Figures and Tables

**Table 1:** Absorption and fluorescence wavelengths of compounds **1h−12h**.

Compound	γ_max_^abs^ nm	γ_max_^em^ (nm)
**1h**	377	469
**2h**	371	459
**3h**	371	460
**4h**	367	459
**5h**	367	460
**6h**	371	460
**7h**	371	460
**8h**	372	462
**9h**	370	461
**10h**	370	460
**11h**	371	459
**12h**	370	459
